# Circular RNA EIF6 (Hsa_circ_0060060) sponges miR-144-3p to promote the cisplatin-resistance of human thyroid carcinoma cells by autophagy regulation

**DOI:** 10.18632/aging.101674

**Published:** 2018-12-12

**Authors:** Feng Liu, Jin Zhang, Long Qin, Ziyao Yang, Jianxia Xiong, Yanyan Zhang, Ruihuan Li, Shujing Li, Huifang Wang, Bo Yu, Wenjun Zhao, Weiran Wang, Zhensu Li, Jing Liu

**Affiliations:** 1 Department of Forensic Medicine, Shanxi Medical University, Taiyuan 030001, Shanxi, China; 2 Department of General Surgery, First Hospital of Shanxi Medical University, Taiyuan 030001, Shanxi, China; 3 Department of General Surgery, First Clinical Medical College of Shanxi Medical University, Taiyuan 030001, Shanxi, China; 4 Department of General Surgery, Second Clinical Medical College of Shanxi Medical University, Taiyuan 030001, Shanxi, China; * Equal contribution

**Keywords:** thyroid carcinoma, circular RNA, Hsa_circ_0060060, miR-144-3p, cisplatin

## Abstract

Anaplastic thyroid carcinoma (ATC) responds for the majority of death of thyroid carcinoma and often causes chemotherapy resistance. We investigated the influence of circEIF6 (Hsa_circ_0060060) on the cisplatin-sensitivity in papillary thyroid carcinoma (PTC) and ATC cells, and explored its regulation to downstream molecules miR-144-3p and Transforming Growth Factor α (TGF-α). Differentially expressed circRNAs in PTC were analyzed using the GSE93522 data downloaded. Expressions of circEIF6, miR-144-3p, TGF-α, autophagy-related proteins and apoptosis-related proteins were determined using qRT-PCR or western blot. RNA pull-down assay and dual luciferase report assay were applied to reveal the target relationships. Autophagy marker LC3 and cell proliferation marker ki67 were evaluated by immunofluorescence and immunohistochemistry. Cell viability was evaluated with MTT assay and cell apoptosis was assessed by flow cytometric analysis. CircEIF6, could promote autophagy induced by cisplatin, thus inhibiting cell apoptosis and enhancing the resistance of PTC and ATC cells to cisplatin. Has-miR-144-3p was the target of circEIF6 and was regulated by circEIF6. Besides, circEIF6 promoted autophagy by regulating miR-144-3p/TGF-α axis, enhancing the cisplatin-resistance in PTC and ATC cells. CircEIF6 promoted tumor growth by regulating miR-144-3p/TGF-α and circEIF6 knock-down enhanced cisplatin sensitivity *in vivo*. CircEIF6 could provide a target for therapy of cisplatin-resistance in thyroid carcinoma.

## Introduction

Thyroid carcinoma is a common cancer, which can be roughly divided into well-differentiated thyroid carcinoma including papillary and follicular thyroid carcinoma, anaplastic thyroid carcinoma (ATC) and medullary thyroid carcinoma (MTC) [1]. However, ATC is an orphan disease with high fatality rate and the effective treatment is not available at present. Generally, these cancers are treated with surgery, radiotherapy, chemotherapy, target therapy, or multimodal therapy [2]. Recently, multi kinase inhibitors (MKIs) as a new drug, show positive activity against receptors of different growth factors, and thus can inhibit tumor cells growth and proliferation. Moreover, MKIs play a function on differentiated thyroid carcinoma [3,4]. Although patients’ condition had some improvements by using those therapies, some patients did not respond to radioiodine (RAI) therapy [5] and occurred drug-resistance, especially in patients with ATC. Due to the undifferentiated phenotype and its aggressive nature of ATC, resistance to conventional treatments such as radiotherapy and chemotherapy, was often observed in the patients with ATC [6], including cisplatin-resistance [7]. Therefore, the patients with ATC have a very poor prognosis.

CircRNAs as the emerging non-coding RNA molecules deleted with a 5'-terminal cap and 3'-terminal poly A tail, were regarded as biomarker and regulators in cancer [8]. Researchers have found that circRNAs could potentially play a virtual role in various cancers. A global reduction of circRNA abundance was found in colorectal cancer and global circRNA abundance was discovered to have a negative correlation to proliferation [9]. CircRNAs regulated tumor progression via multiple mechanisms, such as various signaling pathway [10] and epithelial-mesenchymal transition (EMT) [11,12]. For example, Zhong Z et al. found that circRNA MYLK acted as a competing endogenous RNA for miR-29a and contributed to EMT, promoted bladder cancer development by activating VEGFA/VEGFR2 and Ras/ERK signaling pathway [11]. Similarly, recent studies have shown that circRNAs extensively participate in cell proliferation, cell differentiation, cell autophagy and other biological processes [13]. However, these researches related to circRNAs are not deep in autophagy and drug-resistance.

MiRNAs were also one type of the non-coding RNAs. Numerous studies identified that the inordinate expression of miRNA could promote tumorigenesis [14,15]. Moreover, miRNAs were discovered to be related to the increasing cisplatin-resistance [16] and autophagy, of which miR-146b targeting the PTEN/Akt/mTOR signaling pathway inhibited autophagy in prostate cancer [17]. However, circRNAs function as microRNA (miRNA) sponges and their combination has a regulatory role in transcription and translation [18]. An increasing number of reports have issued some connections between circRNA and miRNA [19]. Nevertheless, the mechanism between the two types of non-coding RNA is limited.

Transforming growth factor-α (TGF-α) is highly associated with certain cancer progression. Previous studies had identified that TGF-α could ally with EGF to promote tumor cell proliferation via various signal [20], such as MEK/VEGF-A-mediated angiogenesis [21]. Furthermore, trans-activator and potential oncogene protein could involve in the regulation of TGF-α on tumor. For instance, Kim JH et al*.* suggested that TGF-α was activated by the hepatitis B viral X protein (HBx) and thus might accelerate hepatocarcinogenesis process [22]. Besides, TGF-α was targeted by the certain miRNA and participated in cancer development. The miR-205 was reported to suppress tumor cell proliferation, invasion, and migration through targeting TGF-α in osteosarcoma [23]. But, there is no research to study the effects of circRNA/miRNA/TGF-α-mediated on cisplatin-resistance in thyroid carcinoma, especially in ATC.

In this study, we used microarray to identify the abnormally expressed circRNAs in papillary thyroid carcinoma (PTC). A highly expressed circEIF6, its target miR-144-3p, and downstream mRNA TGF-α were used to explore the remittance of cisplatin-resistance in PTC and ATC. CircEIF6 sponged miR-144-3p to promote the cisplatin-resistance of human thyroid carcinoma cells by autophagy regulation. The expression of circRNA and miRNA could act as new biomarkers for clinical prognosis and also as new target for drug resistance of cancer therapy.

## RESULTS

### The expression of circEIF6 and miR-144-3p showed an opposite relation in thyroid carcinoma tissues and cells

**Figure 1:**
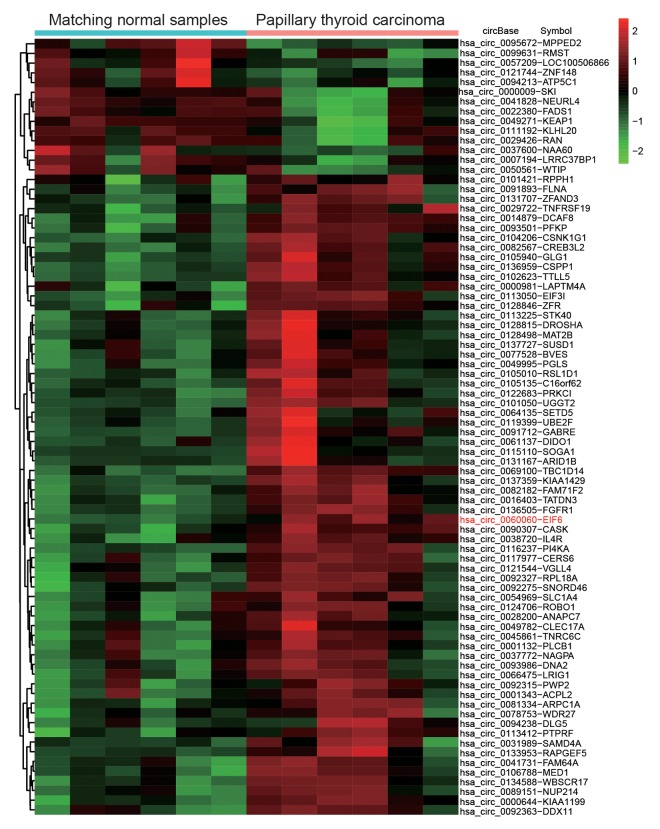
**Differential expression analysis of circular RNAs showed circEIF6 was highly expressed in papillary thyroid carcinoma.** Heat map was generated by differential expression analysis of circular RNAs with GEO data (GEO accession: GSE93522) and revealed circEIF6 was highly expressed in papillary thyroid carcinoma.

**Figure 2:**
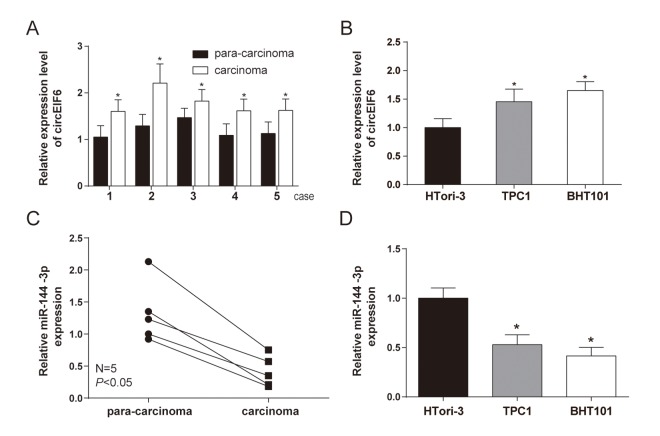
**High level of circEIF6 and low level of miR-144-3p were found in 5 paired anaplastic thyroid carcinoma clinical specimens and thyroid carcinoma cells.** (**A** and **C**) Highly expressed circEIF6 and lowly expressed miR-144-3p in anaplastic thyroid carcinoma tissues were displayed by qRT-PCR. ^*^P < 0.05 compared with the para-carcinoma tissues. (**B** and **D**) High level of circEIF6 and low level of miR-144-3p were also observed in papillary thyroid carcinoma cells (TPC1) and anaplastic thyroid carcinoma cells (BHT101). ^*^P < 0.05 compared with the normal thyroid cells (HTori-3).

CircEIF6 (hsa_circ_0060060) was found highly expressed in PTC through the analysis of gene chips GSE93522 (Figure 1). To further confirm the finding from bioinformatics, the circEIF6 expressions were detected in ATC tissues and both in PTC and ATC cells. Result of qRT-PCR proved that circEIF6 was overexpressed in ATC tissues and both in ATC and PTC cells (TPC1 and BHT101 cells) compared with para-carcinoma tissues and normal thyroid cells (HTori-3), respectively (Figure 2A and 2B, *P* < 0.05). Meanwhile, miR-144-3p expression was evaluated. The results showed miR-144-3p was lowly expressed in ATC tissues and both in TPC1 and BHT101 cells than that in para-carcinoma tissues and HTori-3 cells (Figure 2C and 2D, *P* < 0.05). In brief, the expression of circEIF6 and miR-144-3p showed an opposite connection in thyroid carcinoma tissues and cells.

### CircEIF6 and miR-144-3p targetedly bound in the TPC1 and BHT101 cells with cisplatin treatment

**Figure 3:**
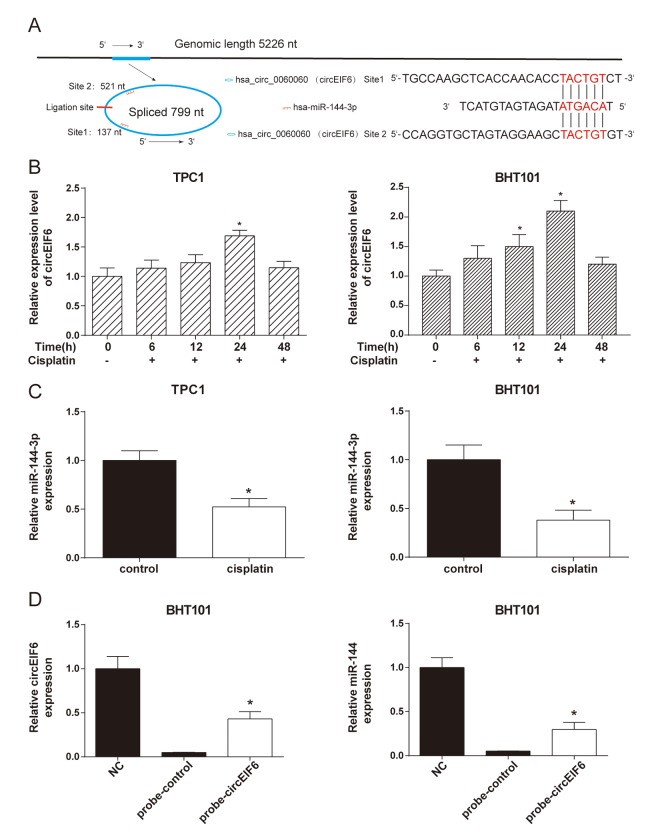
**CircEIF6 and miR-144-3p had a target relationship and a reversed expressed in the TPC1 and BHT101 cells with cisplatin treatment.** (**A**) There are potential 2 target binding sites between miR-144-3p and circEIF6. (**B**) Expressions of circEIF6 were detected in the TPC1 and BHT101 cells with different time cisplatin treatment. ^*^P < 0.05 compared with 0 h. C. The miR-144-3p expressions were detected in the TPC1 and BHT101 cells without or with cisplatin treatment. ^*^P < 0.05 compared with control group. D. The circEIF6 probe successfully increased the circEIF6 and miR-144-3p expressions in TPC1 and BHT101 cells with cisplatin treatment. ^*^P < 0.05 compared with probe-control group.

CircEIF6, originated from 5226 bp genome DNA, circled and formed a circular RNA of 799 nt. The analysis of the circEIF6 and miR-144-3p sequences revealed 2 possible target binding sites between them (Figure 3A). With the treatment of cisplatin (30 μg/ml) in the TPC1 and BHT101 cells, circEIF6 expression was correspondingly changed and had a highest level when treated for 24 h (Figure 3B, *P* < 0.05). Under the treatment with the same concentration of cisplatin for 24 h, miR-144-3p showed a decrease compared with control group without cisplatin treatment in the TPC1 and BHT101 cells (Figure 3C, *P* < 0.05). Moreover, RNA pull-down assay was carried out. Probe of the circEIF6 and control probe were transfected into the TPC1 and BHT101 cells and followed by treatment with cisplatin for 24 h. The circEIF6 probe successfully increased the circEIF6 and miR-144-3p expressions in TPC1 and BHT101 cells with cisplatin treatment (Figure 3C). These findings further supported circEIF6 and miR-144-3p could targetedly bind in thyroid carcinoma cells with cisplatin treatment.

### CircEIF6 regulated TGF-α through targeting miR-144-3p in the TPC1 and BHT101 cells with cisplatin treatment

**Figure 4:**
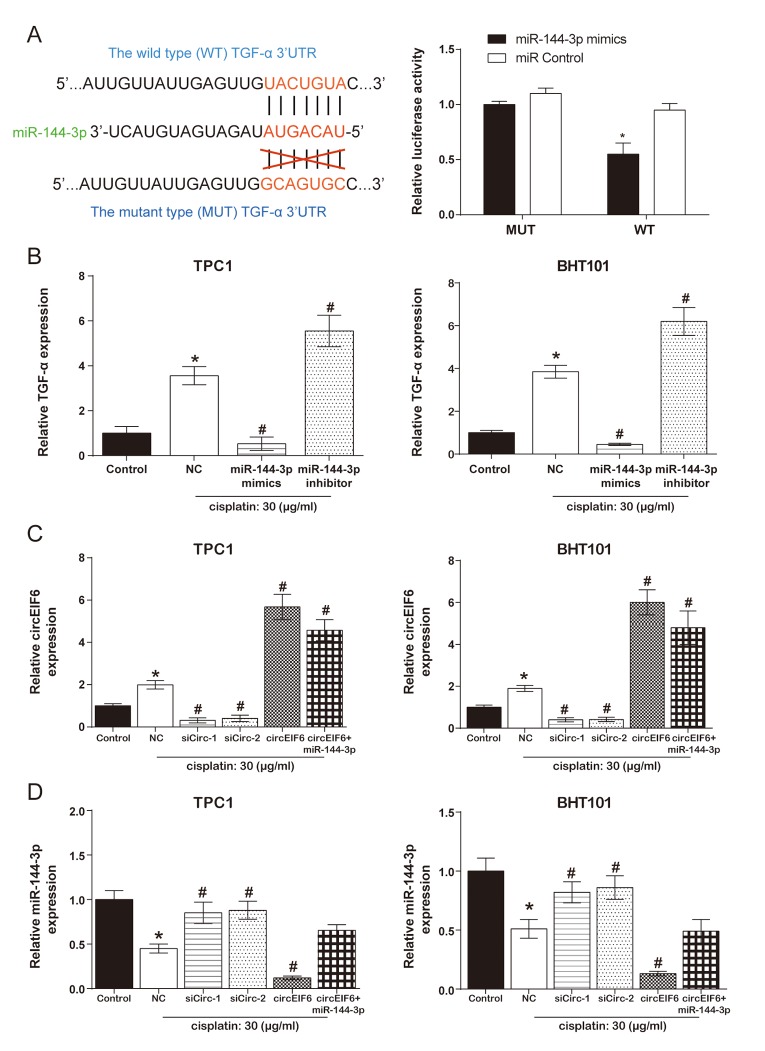
**CircEIF6 regulated TGF-α through targeting miR-144-3p in the TPC1 and BHT101 cells with cisplatin treatment.** (**A**) The target binding site between miR-144-3p and TGF-α was revealed and dual luciferase reporter gene assays was used to verify the target relationship between TGF-α and miR-144-3p (Right). ^*^P < 0.05 compared with WT+miR control group. (**B**) TGF-α mRNA in the TPC1 and BHT101 cells with or without cisplatin treatment was measured after altering miR-144-3p expression and miR-144-3p had an inhibition role on TGF-α under cisplatin treatment. ^*^P < 0.05 compared with control and ^#^*P* < 0.05 compared with NC. (**C**) Transfection efficiency was identified after regulating circEIF6 expression in the TPC1 and BHT101 cells with or without cisplatin treatment. ^*^P < 0.05 compared with control and ^#^*P* < 0.05 compared with NC. (D) MiR-144-3p was decreased by circEIF6 overexpression and increased by circEIF6 knock-down. ^*^P < 0.05 compared with control and ^#^*P* < 0.05 compared with NC.

According to the TargetScan database, position 3527-3533 of TGF-α 3'-UTR has the binding site of miR-144-3p. Meanwhile, in the dual luciferase reporter assays, co-transfection of wild type TGF-α and miR-144-3p mimics significantly suppressed the luciferase activity (Figure 4A). Furthermore, in the TPC1 and BHT101 cells, TGF-α was promoted by cisplatin treatment, was inhibited by miR-144-3p mimics and enhanced by miR-144-3p inhibitor with cisplatin treatment (Figure 4B, *P* < 0.05). Therefore, it was concluded that miR-144-3p was targeted by TGF-α and had a negative regulation on TGF-α.

According to Figure 4C, the expression of circEIF6 both in the TPC1 and BHT101 cells was higher in cisplatin group than that in control group. In addition, under the treatment of cisplatin in the TPC1 and BHT101 cells, circEIF6 expression was obviously suppressed in siCirc-1 and siCirc-2 groups, enhanced in circEIF6 and circEIF6+miR-144-3p groups. However, Figure 4D showed the opposite effects of the above treatments on the expression of miR-144-3p. The conclusion that circEIF6 regulated TGF-α through targeting miR-144-3p in the TPC1 and BHT101 cells with cisplatin treatment could be made.

### CircEIF6 promoted cisplatin-resistance through regulating miR-144-3p/TGF-α in the TPC1 and BHT101 cells

**Figure 5:**
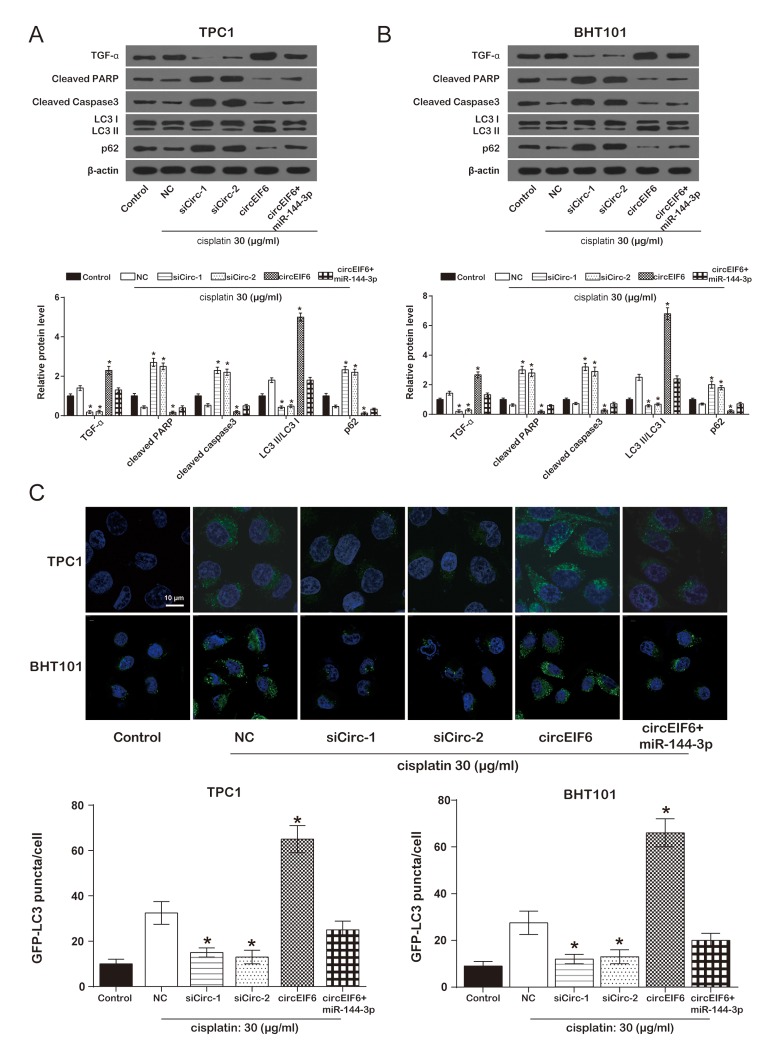
**Overexpression of circEIF6 could enhance autophagy and inhibit apoptosis in the TPC1 and BHT101 cells with cisplatin treatment.** (**A**) Western blot was used to detect the protein of TGF-α, cleaved PPAR, cleaved caspase3, LC3B, p62 expressions. Cleaved PARP and caspase-3 were proteins related to apoptosis; LC3 II/LC3 I ratio and p62 were related to autophagy. ^*^P < 0.05 compared with NC group. (**B**) GFP-LC3 puncta was less in siCirc-1 or siCirc-2 group and more in circEIF6 group after treated with cisplatin, ^*^P < 0.05 compared with NC group.

To study the effect of circEIF6 on cisplatin-resistance in the TPC1 and BHT101 cells, TGF-α, apoptosis-related protein cleaved PARP and cleaved Caspase3, and autophagy-related proteins LC3B and p62 were detected using western blot. In the TPC1 and BHT101 cells with cisplatin treatment, compared with the cisplatin group, the protein levels of cleaved PARP, cleaved Caspase3 and p62 were evidently enhanced by the downregulation of circEIF6 and suppressed by circEIF6 overexpression, while TGF-α protein level and LC3 II/LC3 I ratio were restrained by the downregulation of circEIF6 and increased by circEIF6 overexpression. However, the alteration in circEIF6 group was restored when upregulated miR-144-3p (Figure 5A-5B). In brief, circEIF6 increased TGF-α expression, activated autophagy and inhibited apoptosis. In addition, autophagy assay for GFP-LC3 puncta detection displayed the similar results. As shown in Figure 5C, in the TPC1 and BHT101 cells with cisplatin treatment, GFP-LC3 puncta was less in the siCirc-1 and siCirc-2 groups, nevertheless, GFP-LC3 puncta was significantly more in circEIF6 group, compared with NC group, suggesting that circEIF6 enhanced autophagy.

**Figure 6:**
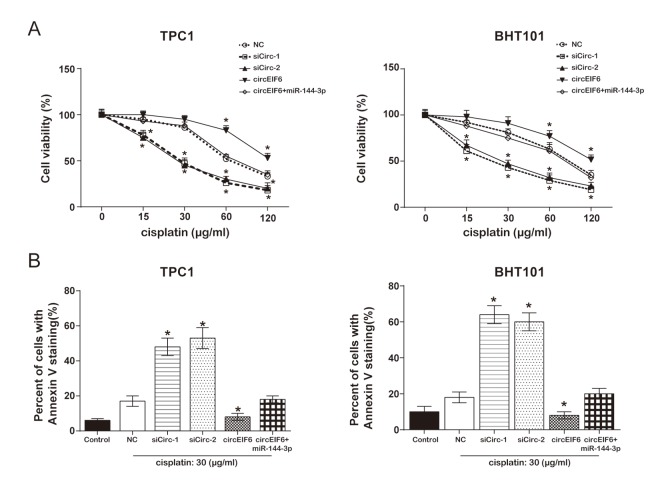
**Overexpression of circEIF6 inhibited apoptosis in the TPC1 and BHT101 cells with cisplatin treatment.** (**A**) MTT assay displayed that in the TPC1 and BHT101 cells with cisplatin treatment, the cell viability in siCirc-1 or siCirc-2 group was significantly inhibited by cisplatin treatment, but was remarkably enhanced by circEIF6 overexpression; miR-144-3p could reverse the function of circEIF6. Cells in control were normalized as 100%. ^*^P < 0.05 compared with the NC group. (**B**) Flow cytometric detected Annexin V staining cells ratio which reflecting the percent of apoptosis cells; less apoptotic cells in circEIF6 overexpression group but more apoptotic cells in circEIF6 knockdown group were observed in the TPC1 and BHT101 cells with cisplatin treatment. ^*^P < 0.05 compared with the NC group.

To in depth explore the effect of circEIF6 on cisplatin-resistance, cell proliferation and apoptosis assays were performed. MTT assay proved that, both in the TPC1 and BHT101 cells with cisplatin treatment, high level circEIF6 promoted cell viability, which was returned to normal level when miR-144-3p was upregulated. However, cell viability was restrained by circEIF6 downregulation (Figure 6A). Flow cytometry displayed that the apoptotic cells were higher in cisplatin+siCirc-1/siCirc-2 group, and lower in cisplatin+circEIF6 group, compared with cisplatin group. However, low level of apoptotic cells inhibited by circEIF6 enhancement was restored by miR-144-3p upregulation (Figure 6B, *P* < 0.05). The above results indicated that circEIF6 could promote cell viability and autophagy but inhibit apoptosis through regulating miR-144-3p/TGF-α. It could be supposed that circEIF6 could promote cisplatin-resistance through regulating miR-144-3p/TGF-αin the TPC1 and BHT101 cells.

### CircEIF6 knock-down enhanced cisplatin sensitivity of BHT101 cells *in vivo*

**Figure 7:**
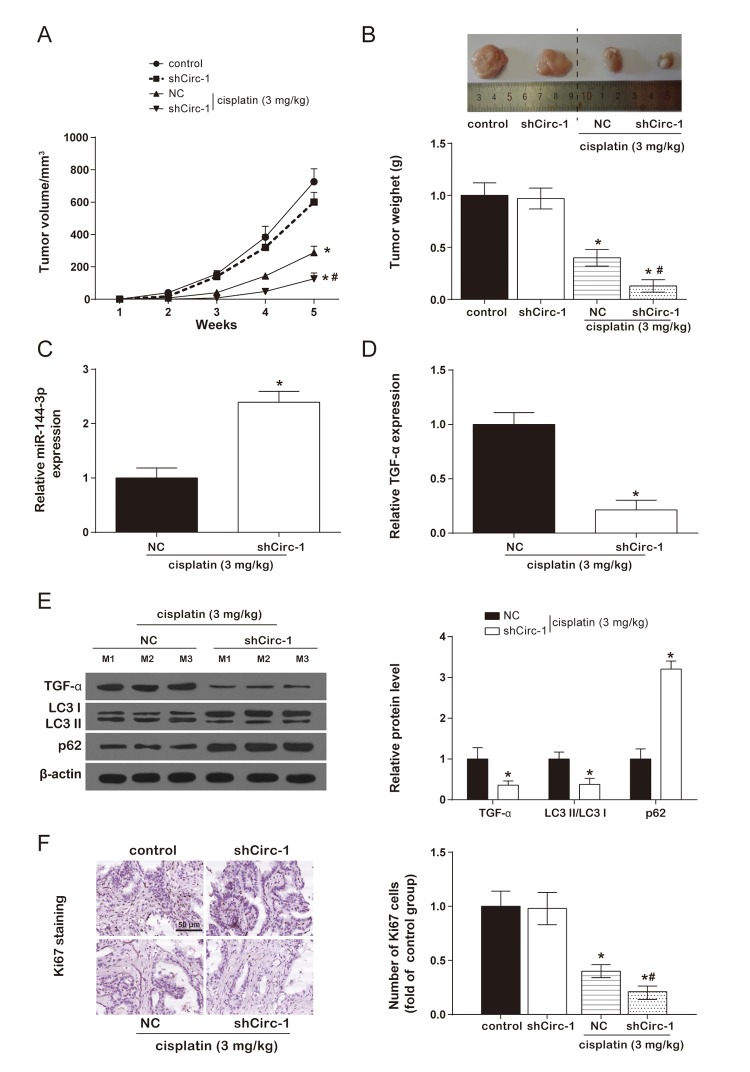
**CircEIF6 knock-down enhanced cisplatin sensitivity of BHT101 cells *in vivo*.** (**A** and **B**) Tumor formation curve and tumor weight quantification showed circEIF6 knockdown played a better anti-tumor effect in cisplatin-treated nude mice. ^*^P < 0.05 compared with control and ^#^*P* < 0.05 compared with NC. (**C** and **D**) Increase of miR-144-3p and reduction of TGF-α were detected in circEIF6 knockdown group with cisplatin treatment. ^*^P < 0.05 compared with NC. (**E**) TGF-α and LC3 II/ LC3 I ratio was lower, while the expression of p62 was much higher in the cisplatin+shCirc-1. ^*^P < 0.05 compared with NC. (**F**) The cell proliferation marker, Ki67, was detected with Immunohistochemistry, which was significantly fewer with cisplatin treatment and was obviously decreased by circEIF6 knock-down under the cisplatin treatment. ^*^P < 0.05 compared with control and ^#^*P* < 0.05 compared with NC.

In the cisplatin group, tumor volume of nude mice was remarkably smaller than control group. However, there was no evident difference between control group and shCirc-1 group. Similarly, circEIF6 knock-down effectively reduced tumor weight in the nude mice with cisplatin treatment, and circEIF6 knock-down had no effect on tumor weight of nude mice (Figure 7A-7B). Meanwhile, miR-144-3p, TGF-α and autophagy-related protein and proliferating cell nuclear antigen ki67 were determined in tumor tissues from nude mice with cisplatin treatment. Results showed circEIF6 knock-down could promote the expression of miR-144-3p and p62 and inhibit TGF-α and ki67 expressions, and the LC3 II/LC3 I ratio (Figure 7C-7F, *P* < 0.05). All above revealed that circEIF6 knock-down could enhance miR-144-3p expression, and inhibit TGF-α expression, autophagy and cell proliferation. Briefly, circEIF6 knock-down enhanced cisplatin sensitivity of BHT101 cells *in vivo*.

## DISCUSSION

ATC as a type of thyroid carcinoma, accounts for small percentage in thyroid carcinoma, however, it is responsible for the majority of death in all thyroid malignancies. Traditional therapies for thyroid carcinoma, such as radioiodine and chemotherapy, could induce the drug resistance, thus contributing to the very poor prognosis in ATC [6]. Recently, several new chemotherapy agents have been applied, such as sorafenib, imatinib, and axitinib, with superimposable activity and response rates of 35%-75%, but the chemoresistance was still not improved [22]. Therefore, making the mechanism of drug-resistance clear is very important. In our study, we identified a number of aberrantly expressed circRNAs in PTC tissue and found that circEIF6 could enhance chemoresistance (cisplatin-resistance) to influence the chemotherapy effects. To figure out the further reason of chemoresistance enhanced by circEIF6, circEIF6, its target miR-144-3p, and downstream mRNA TGF-α were all focused on in PTC and ATC.

Many miRNAs have been reported to twist with circRNAs to regulate biological progress. Memczak S et al*.* revealed the circRNA named CDR1as had a function as a sponge of miR-7, and their data suggested that circRNAs served as post-transcriptional regulators [24]. CircRNAs twisted with targeted miRNAs and participated in a serious of biological progress, such as proliferation and apoptosis, EMT and vascularization, invasion and metastasis [25]. Likewise, we also found that circEIF6 targeting miR-144-3p could influence thyroid carcinoma cell proliferation and cell apoptosis. Detailedly, under the condition of cisplatin treatment, the overexpression of circEIF6 inhibited cell apoptosis and promoted tumor growth with low level of miR-144-3p. Besides, several researches proved that circRNAs functioned through mediating autophagy. CircHECTD1 acted as an endogenous miR-142 sponge to inhibit TIPARP (TCDD inducible poly [ADP-ribose] polymerase) expression with subsequent inhibition of astrocyte activation via autophagy [26]. circRNA.2837 downregulation targeting miR-34 family alleviated sciatic nerve injury through inducing autophagy [27]. Our study was the first to discuss the crosstalk between circEIF6 and miR-144-3p in PTC and ATC, and reveal the promotive role of the circEIF6 in cisplatin-resistance by regulating cell autophagy in PTC and ATC. With the high expression of circRNA, cell autophagy was promoted with cisplatin treatment.

In addition, circRNAs and miRNAs could regulate various signal pathways through the downstream mRNA. For example, circRNA CDR1as promoted osteoblastic differentiation through the miR-7/GDF5/SMAD and p38 MAPK signaling pathway in periodontal ligament stem cells [28]. CircNEK6 targeting miR-370-3p promoted the disease progression by up-regulating FZD8, and activating Wnt signaling pathway in thyroid carcinoma [29]. However, the current study did not discover the signaling pathway of TGF-α downstream and this could be further explored in the future researches. Similarly, circEIF6/miR-144-3p/TGF-α axis was found for the therapy of cisplatin-resistance in current study.

In this study, circEIF6 functioned as a sponger of the miR-144-3p to promote the expression of TGF-α, and enhance cisplatin-resistance by increasing autophagy. MiR-144-3p had been reported in several reports. The expression of miR-144-3p was reported to be low in osteosarcoma and lncRNA TUG1 targeting miR-144-3p promoted tumorigenesis through upregulating EZH2 expression [30]. Moreover, miR-144-3p exerted the anti-tumor effect by targeting c-Met in glioblastoma [31]. Our results were similar to above findings that the expression of miR-144-3p was also decreased in thyroid carcinoma compared with normal thyroid tissue and cell. Meanwhile, TGF-α was negatively correlated with miR-144-3p both in TPC1 and GHT101 cells, and TGF-α overexpression promoted autophagy and suppressed apoptosis. Furthermore, several target miRNAs of TGF-α were identified and participated in disease progress. Of which, miR-505 targeted TGF-α to restrain endometrial cancer [32]. Similarly, miR-205 was found to function as a tumor suppressor in osteosarcoma through targeting TGF-α [23].

Inevitably, some aspects in this study could be improved. For example, the number of ATC tissues was small due to low incidence of ATC. The results from current study could be confirmed in more ATC or PTC tissues. Besides, the downstream signaling pathway could be further explored in the future researches. Otherwise, it is not reported up to now that whether drug-resistance and cell autophagy are co-function. However, our study was first to suppose that circEIF6 and miR-144-3p was associated with chemo-resistance (cisplatin-resistance) by influencing cell autophagy.

## CONCLUSION

As a number of abnormally expressed circRNAs in the PTC tissues were identified, we found that circEIF6 was upregulated and negatively correlated with miR-144-3p. Further experiments showed that circEIF6 could enhance cisplatin-resistance by autophagy activation in TPC1 and BHT101 cells. While the circEIF6 was inhibited, the expression of miR-144-3p was increased and cisplatin-resistance was weaken, as well as TGF-α was declined *in vivo* and *in vitro*.

## MATERIALS AND METHODS

### GEO data

The dysregulation expression data of thyroid carcinoma was downloaded from GSE93522 on GPL19978. The analyzed data included 6 PTC tumors samples and 6 matching contralateral normal samples. Processing of gene expression data was conducted with the Limma package in the R software. RNAs in GSE93522 were considered as significantly differential expression under the conditions of adjusted *P* value < 0.05 and |log_2_FC| > 1.

### Clinical tissue samples

Five paired ATC tissues and para-carcinoma tissues were collected from the First Hospital of Shanxi Medical University during 2012 to 2016. All specimens without chemotherapy or radiotherapy history were confirmed as ATC with pathological department. The specimens were stored in liquid nitrogen for subsequent researches. All the patients signed the written informed consent of their free will. And all the procedures were conducted under the approval of the ethics committee of the First Hospital of Shanxi Medical University.

### Cell culture and reagents

A normal thyroid cell line HTori-3 and two thyroid tumor cell lines TPC1, BHT101 (anaplastic thyroid carcinoma cell) were used for subsequent experiments, which were purchased from BeNa Culture Collection (http://www.bncc.org.cn/) in China. The HTori-3, TPC1 and BHT101 cells were cultured in 90% F-12K medium with 10% FBS, 90% L15 medium with 10% FBS and 80% DMEM medium with 20% FBS, respectively. All mediums contained 100 mg/ml streptomycin and 100 U/ml penicillin. All of the cells were incubated in a humidified condition at 37°C with 5% CO_2_. While the cells reached 70% to 80% confluence, the cells in the logarithmic phase were digested with 0.25% pancreatin for subsequent experiments. Cisplatin was purchased from Sigma-Aldrich (St. Louis, MO, USA). In addition, all cell mediums and other reagents were purchased from Invitrogen (Carlsbad, CA, USA).

### Plasmid construction and cell transfection

The plasmid of pCD2.1-ciR was purchased from Geneseed Biotech (Guangzhou, China) and was combined with circEIF6 sequences to upregulate circEIF6. Short interferon RNAs (si-circ) specific for circEIF6, miR-144-3p mimics, miR-144-3p and control were synthesized by Invitrogen. Cells in the 3^rd^-5^th^ generations were seeded in the 6-well plates at the density of 5×l0^5^ cell/well and then sustained in incubators at 37 ˚C with 5% CO_2_. The Lipofectamine 3000 reagent (Invitrogen, USA) was applied for cell transfection. Transfection procedures were implemented according to the guidance of manufacturer.

### QRT-PCR

The extraction of the total RNA from tissues or cells was performed with TRIzol reagent (Invitrogen, USA). The PrimeScript RT reagent kit (Takara, Tokyo, Japan) and the miRNA First Strand Synthesis Kit (Takara, Japan) were used to perform the reverse transcription for circRNA/mRNA and miRNA, respectively. QRT-PCR was carried out using SYBR Green kit (Thermo Fisher Scientific, Waltham, MA, USA) and Mx3005P QPCR system (Agilent Technologies, Santa Clara, CA, USA). Specific divergent primers for circEIF6 were designed and synthesized by Sangon (Shanghai, China). Meanwhile, circEIF6 and TGF-α were contrasted with β-actin, miRNA was normalized to U6. $2^{-∆∆C_{t}}$ was applied to detect the quantification of circRNA, mRNA and miRNA. Table 1 showed the primer sequences. Each group designed three parallel controls.

**Table 1:** Sequences of primers used for qRT-PCR

	Primers	Sequence
CircEIF6	Sense	5'- GTCAGTGGTGGAGAGTGTCT -3'
	Antisense	5'- AGTAACAAGCTCCGCACGC -3'
TGF-α	Sense	5'- GAGTGACTCACCCGTGGC -3'
	Antisense	5'- CTCACAGTGCTTGCGGAC -3'
β-actin	Sense	5'- GACCTCTATGCCAACACAGTGC -3'
	Antisense	5'- GTACTCCTGCTTGCTGATCCAC -3'
miR-144	Stem-loop Sense	5'- CTCAACTGGTGTCGTGGACTCG GCAATTCAGTTGAGAGTACATC- 3' 5'- CCTCGCACCTGGAGGCTGGCTG -3'
	Antisense	5'- TTATCAGTTGGGAAAATAGTTA -3'
U6	Sense	5'- CTCGCT TCG GCAGCACA -3'
	Antisense	5'- AACGCT TCACGAATTTGCGT -3'

### RNA pull-down assay

Biotin-labeled circEIF6 probe and control probe were synthesized by Sangon Biotech. After treated with cisplatin for 24 h, BHT101 cells were fixed with 1% formaldehyde for 10 minutes and lysed with ultrasound. After centrifugation, the majority of supernatant was removed and kept 50 ul of residues to suspend, which was incubated with the specific circEIF6 probes-streptavidin dynabeads (M-280, Invitrogen) mixture overnight at 30°C. Next day, the mixture of M-280 dynabeads probes-RNAs was washed with 200 µl lysis buffer and incubated with proteinase K to reverse the formaldehyde crosslinking. At last, the specific RNAs in the mixture were extracted using TRizol and detected using qRT-PCR.

### Dual luciferase report gene assay

TaKaRa MutanBEST Kit (TaKaRa, Japan) was used to detect the mutation of TGF-α 3'-UTR following the instructions of manufacturer. The wild-type or mutant-type TGF-α 3'-UTR fragments, miR-144-3p mimics or miR control and the double luciferase reporter plasmid pMIR-GLO were co-transfected into 293T cells using Lipofectamine3000 (Invitrogen). Dual Luciferase Report Assay System (Promega, Madison WI, USA) was employed to measure the luciferase activity according to the manufacturer's protocol.

### Western blot

The total protein of cells or tissues were extracted and prepared for western blot. The BCA Kit (Sigma-Aldrich, USA) was utilized to quantify the protein concentration. By the sodium dodecyl sulfate polyacrylamide gel electrophoresis (SDS-PAGE) electrophoresis, proteins were separated into different bands according to the molecular weight. Subsequently, proteins in different bands were electrophoretically transferred to polyvinylidene fluoride (PVDF) membranes (Millipore, Bedford, MA, USA). Then the membranes were blocked with 5% nonfat milk for 1 h. Primary antibodies (anti-TGF-α, LC3B, p62, cleaved caspase-3, cleaved PARP, β-actin, with different concentration 1:500, 1:3000, 1:1000, 1:500, 1:1000, 1:500, respectivly, BOSTER, Wuhan, China) were incubated at 4˚C overnight. Next day, after membranes were washed with TBST buffer for three times, the corresponding secondary antibodies labelled with the HRP (BOSTER, Wuhan, China) were incubated at room temperature for 2 h. Finally, the signaling was visualized using enhanced chemiluminescence and the relative concentrations were evaluated by Quantity One software (Bio-Rad, Hercules, CA, USA).

### Autophagy assay

Immunofluorescence assay was applied to measure the cell autophagy. The green fluorescent protein (GFP)-LC3 plasmid (Invitrogen) was transfected into the specific treated cells with Lipofectamine 3000 (Invitrogen), which was upregulated or downregulated in the level of circEIF6 or miR-144-3p and were treated with cisplatin for 24 h. Transfection procedures were carried out according to the reagent protocol. After transfection, the cells were fixed with 4% formaldehyde phosphate buffer solution and cell nuclei were stained with DAPI. The green dots in cells were observed by Olympus BX53 fluorescence microscope (Olympus, Tokyo, Japan). Meanwhile, at least 3 wells per group and random 3 horizons per well were observed. The cells were regarded as the autophagy-positive cells with more than 5 green highlights.

### MTT assay

The cells at the density of 5×10^3^ per well were cultured in the 96-well plate under a humidified environment of 37 ˚C and 5% CO_2_. After the cells were treated with specific concentration of cisplatin for 24 h, cells in each well were added with 100 ul culture medium containing 30 μl MTT (Sigma-Aldrich) and incubated for 4 h. Later, the upper medium was carefully removed, and 100 μl dimethyl sulfoxide (DMSO) was added into each well to dissolve the formazan. Lastly, the absorbance value at 490 nm was immediately measured by microplate reader.

### Flow cytometry assay

After cells were successfully transfected, cells were treated with 30 μg/ml cisplatin for 24 h. And then cells were digested by 0.25% trypsin, followed by centrifugation (1000 r/min) at 4 ˚C for 5 min. after the supernatant was removed, cells were washed twice with 1× PBS and then were suspended.

Subsequently, cells were stained with Annexin V-FITC (5 μl) and propidium iodide (PI, 5 ul) in the dark at 37 ˚C for 20 min. Each steps abided by the protocol of apoptosis detection kit (BD Biosciences, San Jose, CA, USA). Finally, the stained cells were quantified using the FACS Aria^TM^ flow cytometer (BD Biosciences, USA).

### Animal experiment

CircEIF6 shRNAs were synthesized (Invitrogen, USA) and inserted into the hU6-MCS-CMV-Puromycin lentiviral vector (Geneseed Biotech) to construct the stable knock-down circEIF6 BHT101 cells. Empty lentiviral vector (Geneseed Biotech) was used as controls. *In vivo* experiments consisted of 4 groups, including empty vector group (control), stable knock-down circEIF6 group (sh-Circ1), co-treatment with cisplatin and empty vector group (NC+cisplatin) and co-treatment with cisplatin and knock-down circEIF6 lentivirus group. BHT101 cells with distinct treatments were subcutaneous injected into 5-week-old male nude mice (obtained from the Laboratory Animal Center of China, Shanghai, China, n = 24). The nude mice in cisplatin groups were treated with 3 mg/kg cisplatin and were delivered medicine every 5 days. The size of the tumors in each group was measured every week based on the formula: volume = length × width^2^ / 2. On week 5, the nude mice were sacrificed. The entire tumors were removed, weighted and used for further experiments. All animals’ experiments were carried out under the support of the Animal Care Committee of the First Hospital of Shanxi Medical University.

### Immunohistochemistry

The excised tumor tissues were fixed using 4% polyoxymethylene, paraffin-embedded and then were sectioned. And then, the sections were incubated with the primary antibodies: anti-Ki67 (1: 67, BOSTER) at 4 ˚C overnight. Later, the sections were added with the corresponding secondary antibodies labeled with horseradish peroxidase (HRP). Finally, after treated with DAB to visualize the Ki67 signal, the sections were observed using microscope.

### Statistical analysis

The data was presented as mean ± SD. GraphPad Prism 6.0 (GraphPad Software, La Jolla, CA) were used to conducted statistical analyses. The significant differences were analyzed using student’s t-test and One-Way ANOVA as appropriate. The statistical significance was confirmed if *P* value was less than 0.05.

### Ethics approval and consent to participate

All procedures performed in studies involving human participants were in accordance with the ethical standards of the First Hospital of Shanxi Medical University institutional committee, and obtained written informed consents from all the participants.
